# Exogenous Oxidative Stress in Human Spermatozoa Induces Opening of the Mitochondrial Permeability Transition Pore: Effect on Mitochondrial Function, Sperm Motility and Induction of Cell Death

**DOI:** 10.3390/antiox13060739

**Published:** 2024-06-18

**Authors:** Anita Bravo, Raúl Sánchez, Fabiola Zambrano, Pamela Uribe

**Affiliations:** 1 Center of Translational Medicine-Scientific and Technological Bioresource Nucleus (CEMT-BIOREN), Faculty of Medicine, Universidad de La Frontera, Temuco 4810296, Chile; anita.bravo@ufrontera.cl (A.B.); raul.sanchez@ufrontera.cl (R.S.); fabiola.zambrano@ufrontera.cl (F.Z.); 2 Department of Preclinical Science, Faculty of Medicine, Universidad de La Frontera, Temuco 4781176, Chile; 3 Department of Internal Medicine, Faculty of Medicine, Universidad de La Frontera, Temuco 4781176, Chile

**Keywords:** exogenous oxidative stress, mitochondrial function, calcium, ATP, PS externalization, DNA fragmentation

## Abstract

Oxidative stress (OS) and disrupted antioxidant defense mechanisms play a pivotal role in the etiology of male infertility. The alterations in reactive oxygen species (ROS) production and calcium (Ca^2+^) homeostasis are the main activators for the mitochondrial permeability transition pore (mPTP) opening. The mPTP opening is one of the main mechanisms involved in mitochondrial dysfunction in spermatozoa. This alteration in mitochondrial function adversely affects energy supply, sperm motility, and fertilizing capacity and contributes to the development of male infertility. In human spermatozoa, the mPTP opening has been associated with ionomycin-induced endogenous oxidative stress and peroxynitrite-induced nitrosative stress; however, the effect of exogenous oxidative stress on mPTP opening in sperm has not been evaluated. The aim of this study was to determine the effect of exogenous oxidative stress induced by hydrogen peroxide (H_2_O_2_) on mPTP opening, mitochondrial function, motility, and cell death markers in human spermatozoa. Human spermatozoa were incubated with 3 mmol/L of H_2_O_2_ for 60 min, and intracellular Ca^2+^ concentration, mPTP opening, mitochondrial membrane potential (ΔΨm), ATP levels, mitochondrial reactive oxygen species (mROS) production, phosphatidylserine (PS) externalization, DNA fragmentation, viability, and sperm motility were evaluated. H_2_O_2_-induced exogenous oxidative stress caused increased intracellular Ca^2+^, leading to subsequent mPTP opening and alteration of mitochondrial function, characterized by ΔΨm dissipation, decreased ATP levels, increased mROS production, and the subsequent alteration of sperm motility. Furthermore, H_2_O_2_-induced opening of mPTP was associated with the expression of apoptotic cell death markers including PS externalization and DNA fragmentation. These results highlight the role of exogenous oxidative stress in causing mitochondrial dysfunction, deterioration of sperm motility, and an increase in apoptotic cell death markers, including PS externalization and DNA fragmentation, through the mPTP opening. This study yielded new knowledge regarding the effects of this type of stress on mitochondrial function and specifically on mPTP opening, factors that can contribute to the development of male infertility, considering that the role of mPTP in mitochondrial dysfunction in human sperm is not completely elucidated. Therefore, these findings are relevant to understanding male infertility and may provide an in vitro model for further research aimed at improving human sperm quality.

## 1. Introduction

Infertility is the inability to conceive after one year of regular unprotected intercourse [1]. It is a highly prevalent pathology [2], and ranks third in frequency of diagnosis after cancer and cardiovascular/cerebrovascular pathologies [3]. Around 15% of couples worldwide are affected by infertility, and each year, over seven million couples require assistance to achieve a successful pregnancy [4]. Worldwide, 50% of infertility cases can be attributed to the male factor [5,6], and in Latin America, the male factor contributes to 52% of infertility cases [7]. Medical conditions, lifestyle, and environmental factors [8,9,10,11,12] are often associated with male infertility. However, in most cases, the etiology is unknown and is classified as idiopathic male infertility [13,14]. Oxidative stress (OS) has been extensively studied and is now widely recognized as a significant contributor to male infertility [5,15,16,17,18,19,20,21,22,23,24]. In fact, it has been identified as a cause or mechanism for idiopathic male infertility [25,26,27]. To explain the possible etiology of male infertility in infertile men with abnormal semen characteristics and OS, the term male oxidative stress infertility (MOSI) has been proposed [6].

Sperm mitochondria play an important role in cell metabolism and energy production through ATP synthesis [28,29], and other important cellular processes, including the regulation of calcium (Ca^2+^) homeostasis, cell death processes, and the generation of reactive oxygen species (ROS) [28,30]. Mitochondria are the main source of intracellular ROS production in sperm, and physiological levels of ROS represent important molecules involved in processes such as motility, hyperactivation, capacitation, acrosome reaction, and fertilization [31]. However, the imbalance in favor of pro-oxidant species and against the antioxidant capacity can disrupt redox signaling and/or cause molecular damage, resulting in OS [32]. OS has been linked to male infertility in human spermatozoa [33,34]; therefore, due to the critical role of mitochondria in sperm function, any alteration in mitochondrial activity can cause OS and lead to male infertility [35,36]. The mitochondrial permeability transition pore (mPTP) is a high-conductance channel located at the contact sites between the inner mitochondrial membrane and the outer mitochondrial membrane [37,38], and is responsible for the integration of oxidative phosphorylation (OXPHOS) for energy production and induction of cell death when it is converted into a nonspecific channel [39,40]. OS and alterations in Ca^2+^ homeostasis are the main activators of mPTP opening in somatic cells [41,42]. This mechanism is characterized by an increase in ROS production associated with OS, which alters intracellular Ca^2+^ homeostasis, generates mitochondrial Ca^2+^ overload, alters mitochondrial pH, and increases ROS generation in the mitochondrial respiratory chain [43,44]. Opening of the mPTP increases the permeability of the inner mitochondrial membrane to solutes up to 1.5 kDa, leading to a dissipation of the mitochondrial membrane potential (ΔΨm) and the release of Ca^2+^ and ROS [45]. If the opening of this pore is transient, physiological functions are fulfilled by the release of Ca^2+^ and ROS; on the contrary, if the opening is prolonged, there is a rapid collapse of ΔΨm, a decrease in ATP levels and a breakdown of the outer mitochondrial membrane [30], events that lead to the release of mitochondrial pro-apoptotic factors, cytochrome C, the activation of caspases and finally the activation of apoptosis [46,47,48,49,50]. The ability of H_2_O_2_ to induce exogenous oxidative stress and mobilize Ca^2+^ from intracellular stores and induce cell death by mPTP opening has been demonstrated in various cell types, such as mouse pancreatic acinar cells [51], human platelets [52], neutrophils [53], and rat hippocampal astrocytes [54]. In human spermatozoa, H_2_O_2_ is a powerful ROS capable of generating highly toxic effects on sperm function under in vitro conditions [55,56,57,58,59,60] and also induces the expression of some apoptotic markers such as activated caspase-3, PS externalization, and DNA fragmentation [61]. Also, it stimulates the mobilization of Ca^2+^ from intracellular reserves, increasing the concentration of cytosolic Ca^2+^, which in turn, stimulates the accumulation of this ion in the mitochondria of spermatozoa and induces the activation of caspases 3 and 9, and PS externalization, which requires the mobilization and entry of Ca^2+^ into the mitochondria [62]. In somatic cells, if this increase in mitochondrial Ca^2+^ is sustained over time, Ca^2+^ accumulates in the mitochondrial matrix, leading to mitochondrial Ca^2+^ overload, causing inflammation of the mitochondrial matrix, opening of the mPTP [63] and release of pro-apoptotic molecules, leading to cell death [48]. Thus, opening of the mPTP may be related to exogenous oxidative stress induced by exposure to H_2_O_2_ in somatic cells.

In spermatozoa, mPTP opening is one of the main mechanisms involved in mitochondrial dysfunction, which induces a state of OS and negatively affects energy supply, motility and fertility [40,64]. In addition, mPTP opening in human spermatozoa has been associated with ionomycin-induced endogenous oxidative stress [65] and peroxynitrite-induced nitrosative stress [66]. It is important to emphasize that spermatozoa can be exposed to various sources of exogenous oxidative stress that impair sperm function through the generation of ROS, including various environmental factors, diseases such as varicocele, lifestyle, and the production of ROS by leukocytes in the male and female genital tract [67,68,69,70]. Although spermatozoa are exposed to exogenous oxidative stress under various conditions during their maturation and/or transit through the female genital tract, the effects of this type of stress on mitochondrial function and specifically on mPTP opening have not been demonstrated. The aim of this study was to evaluate the effect of exogenous oxidative stress induced by H_2_O_2_ on mPTP opening, mitochondrial function, cell death markers and motility in human spermatozoa.

## 2. Materials and Methods

### 2.1. Semen Collection and Analysis

In the present study, the semen samples were obtained from eight normozoospermic healthy donors. The donors were students of the Universidad de La Frontera, Temuco, Chile, between the ages of 19 and 34 years, with no associated chronic diseases. Donors were previously informed of the purpose of the study, signed an informed consent form approved by the Scientific Ethics Committee of the Universidad de La Frontera, and provided more than one sample during the study. Semen collection and analysis were performed according to the guidelines of the World Health Organization (WHO) [71], and the semen samples used in this study met the WHO criteria for normality. The motile sperm population was then selected from the semen samples using the swim-up technique [72]. A medium human tubal fluid (HTF medium; [73]) was used.

### 2.2. Experimental Design

The in vitro activation of mPTP opening, the effect on the expression of cell death markers and the alteration of sperm function were studied in selected human spermatozoa under conditions of exogenous oxidative stress induced by exposure to H_2_O_2_. For this purpose, spermatozoa were incubated with H_2_O_2_ according to previously described experimental conditions established in our laboratory to generate an exogenous oxidative stress [74]. Briefly, this was achieved by incubating the spermatozoa with 3 mmol/L of H_2_O_2_ for 60 min [74]. After incubation, the treated spermatozoa and their respective untreated controls were washed once by centrifugation at 500× *g* for 5 min and suspended in 1 mL of HTF medium. Then, mPTP opening was evaluated (see below) along with the following markers associated with this event: intracellular Ca^2+^ concentration, ΔΨm, ATP levels, and mROS production. PS externalization, DNA fragmentation and viability were then assessed to determine the expression of cell death markers. Sperm motility was also evaluated as a marker of sperm function. Five independent experiments for each variable were performed on different days with different semen samples, and the experiments were performed in duplicate.

### 2.3. Evaluation of mPTP Opening in Human Spermatozoa Exposed to H_2_O_2_-Induced Exogenous Oxidative Stress

The opening of the mPTP was evaluated using the Mitoprobe Transition Pore Assay Kit (Molecular Probes, Invitrogen, Eugene, OR, USA), which has been previously used on somatic cells [75] and on human spermatozoa [65,66]. This method monitors the opening of mPTP by passive diffusion of the calcein-AM stain into the cell. This stain accumulates in all cytosolic compartments, including mitochondria. However, the addition of Cobalt chloride (CoCl_2_) can quench the fluorescence of cytosolic calcein but maintain the fluorescence of mitochondrial calcein because CoCl_2_ cannot pass through the intact IMM. When the mPTP opening occurs, the permeability of the mitochondrial membranes is altered, causing a drastic decrease in the fluorescence of mitochondrial calcein [75]. For the experiments, 2 × 10^6^ spermatozoa/mL previously incubated with H_2_O_2_ were exposed to 0.01 μmol/L of calcein-AM and 0.4 μmol/L of CoCl_2_ for 15 min at 37 °C. Three methodological controls were included as recommended by the manufacturer: (i) 0.01 μmol/L of calcein-AM; (ii) 0.01 μmol/L of calcein-AM and 0.4 μmol/L of CoCl_2_; and (iii) 0.01 μmol/L of calcein-AM, 0.4 μmol/L of CoCl_2_ and 0.5 μmol/L of ionomycin. After incubation, spermatozoa were washed, suspended in 300 μL of HTF, and incubated with 1 mmol/L of propidium iodide (Sigma-Aldrich, Inc., St. Louis, MO, USA). Finally, the results were acquired by flow cytometry and expressed as mean fluorescence intensity (MFI) of calcein-AM (see Analysis by flow cytometry below).

### 2.4. Evaluation of Intracellular Ca^2+^ Concentration in Human Spermatozoa Exposed to H_2_O_2_-Induced Exogenous Oxidative Stress

Measurement of intracellular Ca^2+^ concentration was performed using the Fluo4-AM probe (Molecular Probes, Life Technologies, Carlsbad, CA, USA), a photostable probe with low phototoxicity [76]. For this analysis, 2 × 10^6^ spermatozoa/mL were incubated with Fluo4-AM (5 μmol/L) for 45 min at 37 °C [77]. Then, the aliquots were washed by centrifugation at 500× *g* for 5 min and resuspended in 1 mL of HTF, which was deposited in a quartz cuvette. Subsequently, an additional 1 mL of HTF was added, and the spermatozoa were incubated with 3 mmol/L of H_2_O_2_ for 60 min (3600 s) at 37 °C. The relative fluorescence units (RFU) of Fluo4-AM were measured using a fluorimeter (Photon Technology International PTI, Delta RAM X) by conventional fluorometry [78]

### 2.5. Evaluation of ΔΨm in Human Spermatozoa Exposed to H_2_O_2_-Induced Exogenous Oxidative Stress

ΔΨm was assessed using tetramethylrhodamine methyl ester perchlorate (TMRM; Sigma-Aldrich Inc., St. Louis, MO, USA), a lipophilic and nontoxic dye capable of accumulating in mitochondria in proportion to the state of ΔΨm [79]. Sperm viability was simultaneously analyzed by incubation with the fluorescent dye SYTOX^TM^ green (Molecular Probes, ThermoFisher, MA, USA). Briefly, 2 × 10^6^ spermatozoa/mL previously exposed to H_2_O_2_ and an untreated control, were washed by centrifugation and suspended in 1 mL of HTF. The spermatozoa were immediately incubated for 15 min at 37 °C with TMRM at 250 μmol/L and SYTOX^TM^ green at 50 μmol/L. After this time, the spermatozoa were washed twice and resuspended in 300 μL of HTF. The results were obtained by flow cytometry (Becton, Dickinson and Company, BD Biosciences, San Jose, CA, USA) and expressed as MFI of TMRM (see Analysis by flow cytometry below).

### 2.6. Evaluation of ATP Levels in Human Spermatozoa Exposed to H_2_O_2_-Induced Exogenous Oxidative Stress

The ATP Determination Kit (Molecular Probes, Life Technologies, Carlsbad, CA, USA) was used to analyze ATP levels according to the manufacturer’s instructions. This kit uses a bioluminescence assay for the quantitative determination of ATP with recombinant firefly luciferase and its substrate, D-luciferin, and is based on the requirement of luciferase for ATP to produce light from the reaction. For this, 5 × 10^6^ spermatozoa/mL previously exposed to H_2_O_2_ and untreated controls were washed by centrifugation and suspended in 1 mL of HTF. Immediately, 10 µL of the cell suspension was added to a 96-well white wall luminometer plate containing 100 μL of the standard reaction solution (deionized H_2_O, 20× reaction buffer, DTT, D-luciferin and recombinant luciferase) per well and incubated for 60 s at 25 °C. At the end, the relative luminescence units (RLU) were measured in a luminometer (Luminoskan, Thermo Scientific, Atlanta, GA, USA), and the background luminescence was subtracted in each determination.

### 2.7. Evaluation of Mitochondrial Superoxide Anion Content in Human Spermatozoa Exposed to H_2_O_2_-Induced Exogenous Oxidative Stress

The mROS production was evaluated using the MitoSOX^TM^ red probe (Molecular Probes, Life Technologies, Carlsbad, CA, USA). The MitoSOX^TM^ red exclusively enters the mitochondria and reveals a red fluorescent product of oxidation by superoxide anion. MitoSOX red was used in combination with SYTOX^TM^ green (Molecular Probes, Life Technologies, Carlsbad, CA, USA) to assess cell viability. Briefly, 2 × 10^6^ spermatozoa/mL previously incubated with H_2_O_2_ and untreated controls, were washed once by centrifugation and suspended in 1 mL of HTF. The cells were then incubated for 20 min at 37 °C with 3 μmol/L of MitoSOX^TM^ red and 0.08 μmol/L of SYTOX^TM^ green. After incubation, the cells were resuspended in 300 μL of HTF and analyzed using flow cytometry, and the results were expressed as the MFI of MitoSOX^TM^ red (see Analysis by flow cytometry below).

### 2.8. Evaluation of PS Externalization in Human Spermatozoa Exposed to H_2_O_2_-Induced Exogenous Oxidative Stress

PS externalization was assessed using the Dead Cell Apoptosis Kit with Annexin V Alexa Fluor™ 488 & Propidium Iodide (PI) kit (Molecular Probes, Life Technologies, Carlsbad, CA, USA), which has been previously used in human spermatozoa, according to the manufacturer’s instructions [74]. Briefly, spermatozoa resuspended in 100 μL of 1X Annexin V binding buffer were incubated with 2 μL of Alexa Fluor^®^ 488 Annexin V and 1 μL of PI (100 μg/mL) for 15 min at room temperature. After incubation, 400 μL of 1X Annexin V binding buffer was added, and the results were analyzed by flow cytometry (see Analysis by flow cytometry below). The results were expressed as the percentage of sperm positive for Annexin V (cells with PS externalization) and negative for PI (live cells).

### 2.9. Evaluation of DNA Fragmentation in Human Spermatozoa Exposed to H_2_O_2_-Induced Exogenous Oxidative Stress

DNA fragmentation was analyzed by the modified terminal deoxynucleotidyl transferase-mediated dUTP nick-end labeling (TUNEL) assay [80], using the In Situ Cell Death Detection Kit, Fluorescein (Roche, Mannheim, Germany) with FITC-labeled dUTPs. For this, 2 × 10^6^ spermatozoa/mL previously incubated with H_2_O_2_, an untreated control, and a positive control treated with DNAse I were washed and, to unpack the DNA, the cells were incubated with 500 µL of 2 µmol/L of dithiothreitol for 30 min at room temperature [80]. Subsequently, the spermatozoa were washed, fixed in 200 µL of 2% paraformaldehyde for 15 min at 4 °C, and permeabilized with 0.2% Triton X-100 for 5 min at room temperature. After permeation, the cells were incubated with 50 µL of TUNEL reaction solution for 60 min at 37 °C. Then, 800 μL of HTF and 1 mmol/L of PI (Sigma-Aldrich, Inc., St. Louis, MO, USA) were added and incubated for 5 min at room temperature. The cells were then washed and resuspended in 300 μL of HTF, and the percentage of DNA fragmentation was determined by flow cytometry. The results were expressed as the percentage of TUNEL FITC-positive spermatozoa (see Analysis by flow cytometry below).

### 2.10. Evaluation of Sperm Motility in Human Spermatozoa Exposed to H_2_O_2_-Induced Exogenous Oxidative Stress

Sperm motility was evaluated using the CASA system with the Integrated Sperm Analysis System software version 1 (ISAS; Proiser, Valencia, Spain). Briefly, 5 × 10^6^ spermatozoa/mL were exposed to 3 mmol/L of H_2_O_2_ for 60 min at 37 °C. Then, 2 μL of spermatozoa was placed in Leja counting chambers and observed under a microscope with a stage tempered at 37 °C. A minimum of 100 spermatozoa from at least four different fields were analyzed. An untreated control was included. Finally, the percentage of total sperm motility was evaluated using the CASA system.

### 2.11. Analysis by Flow Cytometry

A BD FACSCanto II flow cytometer (Becton, Dickinson and Company, BD Biosciences, San Jose, CA, USA), controlled by FACSDivaTM 6.1.3 software (Becton, Dickinson and Company), was used for fluorescence analysis. Analyses were performed on logarithmic scales, and a total of 10 000 events were acquired in each experiment. The fluorophores were excited at 488 nm with an argon laser. The green fluorescence of calcein-AM, Alexa Fluor^®^ 488, SYTOX Green and FITC was read with a bandpass filter of 530/30 nm. The red fluorescence of TMRM, MitoSox red and PI-positive was read with a bandpass filter of 585/42 nm.

### 2.12. Statistical Analysis

Statistical analysis was performed using the GraphPad Prism 5 software package (GraphPad, La Jolla, CA, USA). D’Agostino’s test was used to check the normality of the data, and numerical results that did not pass the normality test were transformed to a logarithmic scale. Results were expressed as mean ± standard deviation (SD). For the statistical analysis of ΔΨm, ATP levels, mROS production, PS externalization, DNA fragmentation, and viability, a t-test of paired samples was used to compare against their respective untreated controls. One-way analysis of variance (ANOVA) with Dunnett’s post-test was used for statistical analysis of mPTP opening. Two-way ANOVA with Bonferroni’s post-test was used for statistical analysis of intracellular Ca^2+^ concentration. One-way ANOVA for nonparametric data (Kruskal–Wallis test) with Dunn’s post-test was used for statistical analysis of total sperm motility. A *p* value below 0.05 was considered statistically significant. All experiments were performed in duplicate, and five independent experiments were performed on different days with different semen samples.

## 3. Results

### 3.1. Analysis of mPTP Opening in Human Spermatozoa Exposed to Exogenous Oxidative Stress

**Figure 1:**
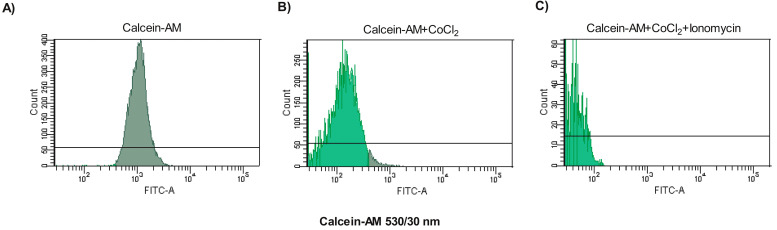
Analysis of the mitochondrial permeability transition pore (mPTP) opening in human spermatozoa with three methodological controls by flow cytometry. The images correspond to representative histograms of the MFI of Calcein-AM from the flow cytometric analysis of a single sperm sample. (**A**) Spermatozoa incubated with calcein-AM alone show high levels of fluorescence. (**B**) Co-incubation of sperm with cobalt chloride (CoCl_2_) causes calcein-AM quenching except in the mitochondrial matrix, because the intact inner membrane does not allow cobalt chloride entry. (**C**) Sperm incubated with ionomycin show mPTP opening, CoCl_2_ entry and calcein quenching in the matrix.

The results showed that in the Cal group, the fluorescence of the cells was the highest (1418.0 ± 567.9), consistent with the accumulation of calcein in all cytosolic compartments, including the mitochondria (Figure 1A). In the Cal + Co group, the fluorescence of calcein was decreased (240.1 ± 65.9) by the addition of the quencher CoCl_2_, but since it could not cross the intact inner mitochondrial membrane, CoCl_2_ only quenched the fluorescence of cytosolic calcein, leaving the fluorescence of mitochondrial calcein intact (Figure 1B). In the control group, Cal + Co + Io, when the opening of the mPTP occurred, the permeability of the mitochondrial membranes was altered, causing a drastic decrease (52.6 ± 0.9) in the fluorescence of calcein (Figure 1C).

**Figure 2:**
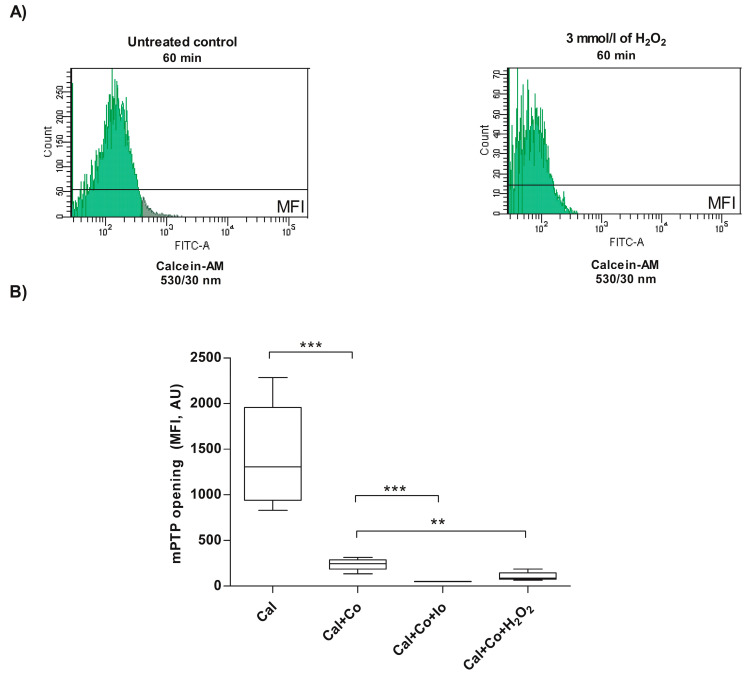
Evaluation of mPTP opening in human spermatozoa exposed to H_2_O_2_-induced exogenous oxidative stress. For all experiments, spermatozoa were exposed to 3 mmol/L of H_2_O_2_ for 60 min at 37 °C, and the results correspond to the mean ± SD of five different experiments. ** *p* < 0.01, *** *p* < 0.001. (**A**) The images correspond to representative histograms of the MFI of Calcein-AM from the flow cytometric analysis of one experiment. (**B**) The mPTP opening was evaluated with the calcein-AM/CoCl_2_ method. Three method controls were also included: Calcein-AM (Cal), corresponding to whole cell fluorescence, Calcein-AM + CoCl_2_ (Cal + Co), indicating intact mitochondrial fluorescence, and Calcein-AM + CoCl_2_ + ionomycin (Cal + Co + Io), showing that the fluorescence decreased due to the ruptured mitochondrial membrane after ionomycin-induced mPTP opening. MFI, mean fluorescence intensity; AU, arbitrary units; H_2_O_2_, hydrogen peroxide; Io, ionomycin.

In the experimental group consisting of spermatozoa treated with H_2_O_2_, the results showed that the fluorescence of calcein was reduced (106.0 ± 48.1) compared to the untreated control (240.0 ± 65.9) (Cal + Co), indicating that under these experimental conditions, H_2_O_2_ induces mPTP opening (*p* < 0.01; Figure 2A,B).

### 3.2. Analysis of Intracellular Ca^2+^ Concentration in Human Spermatozoa Exposed to Exogenous Oxidative Stress

**Figure 3:**
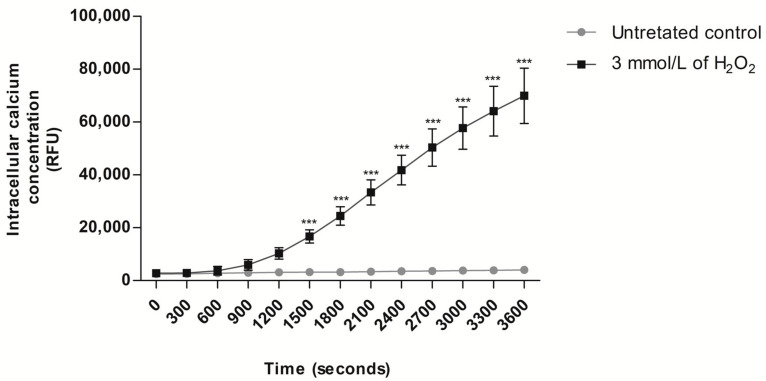
Evaluation of intracellular Ca^2+^ concentration in human spermatozoa exposed to H_2_O_2_-induced exogenous oxidative stress. For all experiments, spermatozoa were exposed to 3 mmol/L of H_2_O_2_ for 60 min at 37 °C, and the results correspond to the mean ± SD of five different experiments. *** *p* < 0.001. The intracellular Ca^2+^ concentration was evaluated with the conventional fluorimetry method. An untreated control was included. AU, arbitrary units; RFU, relative fluorescence units; H_2_O_2_, hydrogen peroxide.

To verify the ability of H_2_O_2_-induced exogenous oxidative stress to mobilize Ca^2+^ from intracellular stores and increase intracellular Ca^2+^ concentration, a condition that directly triggers the mPTP opening [81], intracellular Ca^2+^ levels were measured after exposure to H_2_O_2_. The results showed that the exposure of human spermatozoa to H_2_O_2_ caused an increase in the relative fluorescence intensity of Fluo4-AM compared to the untreated control (*p* < 0.001; Figure 3), starting from 25 min (1500 s) (untreated control: 3210.2 ± 1034.0 and H_2_O_2_ treatment: 16,698.4 ± 2507.0) and lasting for up to 60 min (3600 s) (untreated control: 4004.0 ± 1186.0 and H_2_O_2_ treatment: 69,922.7 ± 10,485.0) of exposure to H_2_O_2_.

### 3.3. Analysis of Mitochondrial Changes in Human Sperm Cells Exposed to Exogenous Oxidative Stress

**Figure 4:**
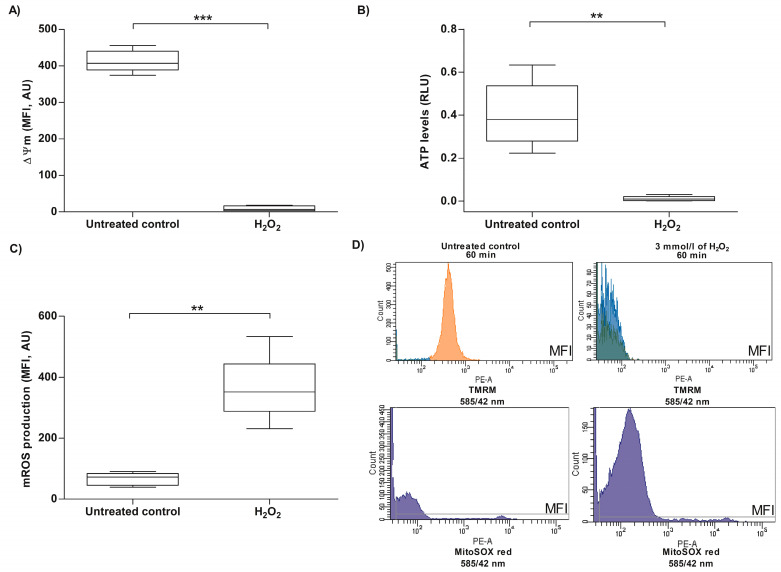
Evaluation of mitochondrial changes in human sperm cells exposed to exogenous oxidative stress. For all experiments, spermatozoa were exposed to 3 mmol/L of H_2_O_2_ for 60 min at 37 °C, and the results correspond to the mean ± SD of five different experiments. ** *p* < 0.01, *** *p* < 0.001. (**A**) The ΔΨm was evaluated using TMRM stain. An untreated control was included. (**B**) The ATP levels were evaluated with the bioluminescence assay method. An untreated control was included. (**C**) Mitochondrial ROS (mROS) production was evaluated using the MitoSOX red probe. An untreated control was included. (**D**) The images correspond to representative histograms of the MFI of TMRM and MitoSOX red from the flow cytometric analysis of one experiment. MFI, mean fluorescence intensity; AU, arbitrary units; RLU, relative luminescence units; H_2_O_2_, hydrogen peroxide.

In somatic cells, opening of the mPTP is associated with several changes at the mitochondrial level, including changes in ΔΨm, ATP levels and mROS production [81]; they were evaluated in human spermatozoa exposed to H_2_O_2_, under the same experimental conditions that triggered the mPTP opening and induced an increase in intracellular Ca^2+^ levels. The analysis of ΔΨm in selected human spermatozoa, exposed to H_2_O_2_ for 60 min, revealed that the MFI of TMRM was significantly decreased (9.4 ± 7.2) compared to the untreated control (413.3 ± 29.8), indicating that H_2_O_2_ also induced a ΔΨm dissipation in human spermatozoa (*p* < 0.001; Figure 4A–D). Consistent with this, the ATP levels analyzed in spermatozoa incubated with H_2_O_2_ showed a decrease in the relative luminescence units of ATP (0.0113 ± 0.0119) compared to the untreated control (0.40 ± 0.15), indicating that under the experimental conditions tested, H_2_O_2_ induces a decrease in ATP content in human spermatozoa (*p* < 0.01; Figure 4B). Regarding mROS production, the results showed an increase in MFI of MitoSOX red (363.0 ± 108.2) compared to the untreated control (65.8 ± 20.5) in human spermatozoa incubated with 3 mmol/L of H_2_O_2_, indicating that the treatment with H_2_O_2_ caused an increase in mROS production (*p* < 0.01; Figure 4C,D). Exogenous oxidative stress-induced mPTP opening is associated with impairment of mitochondrial function in human spermatozoa.

### 3.4. Effect of H_2_O_2_-Induced Exogenous Oxidative Stress on Sperm Motility of Human Spermatozoa

**Figure 5:**
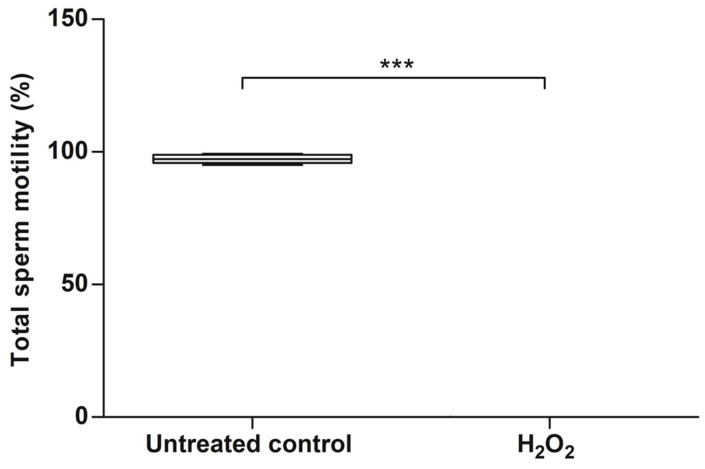
Effect of H_2_O_2_-induced exogenous oxidative stress on sperm motility. Selected human spermatozoa were exposed to 3 mmol/L of H_2_O_2_ for 60 min at 37 °C, and the results correspond to the mean ± SD of five different experiments. *** *p* < 0.001. H_2_O_2_, hydrogen peroxide.

Another critical parameter of sperm functionality is sperm motility, which was evaluated under the same experimental conditions. The results showed a drastic decrease (0.0% ± 0.0) in total sperm motility, which was significant after 60 min of incubation with 3 mmol/L of H_2_O_2_, compared to the untreated control (97.4% ± 1.93; *p* < 0.001; Figure 5).

### 3.5. Analysis of Apoptotic Cell Death Markers on Human Sperm Cells Exposed to Exogenous Oxidative Stress

**Figure 6:**
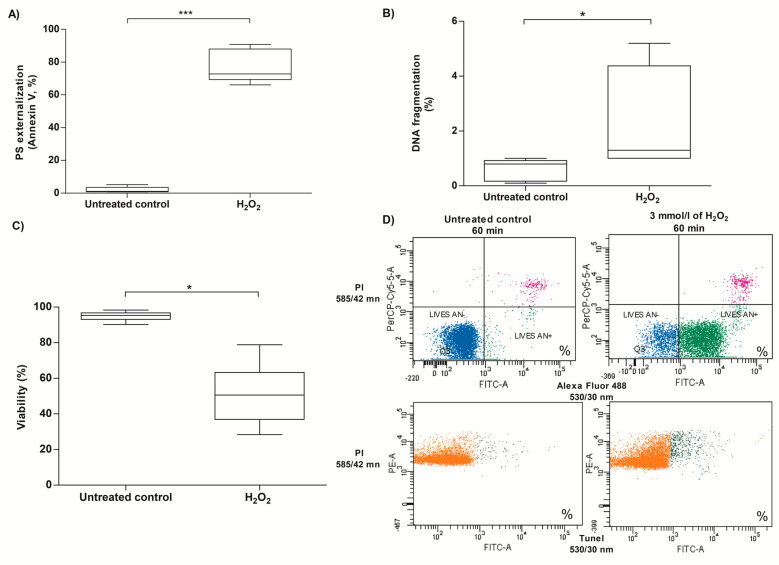
Effect of H_2_O_2_-induced exogenous oxidative stress on the expression of cell death markers. (**A**) Phosphatidylserine (PS) externalization, (**B**) DNA fragmentation, (**C**) sperm viability, and (**D**) representative dot plot of PS externalization (viable cells, lower left quadrant highlighted in blue; early apoptotic cells, bottom right quadrant highlighted in green; late apoptotic cells, top right quadrant highlighted in purple) and DNA fragmentation (non-viable cells, highlighted in orange; TUNEL FITC-positive spermatozoa, highlighted in green) from the flow cytometric analysis of one experiment. For this experiment, spermatozoa were exposed to 3 mmol/L of H_2_O_2_ for 60 min at 37 °C, and the results correspond to the mean ± SD of five different experiments. * *p* < 0.05, *** *p* < 0.001. H_2_O_2_, hydrogen peroxide; PS, phosphatidylserine; Live AN^-^, viable cells; Live AN^+^, early apoptotic cells.

Opening of the mPTP in somatic cells can activate several cell death mechanisms, including apoptotic responses [81]. Two different apoptotic markers, PS externalization and DNA fragmentation, along with viability, were analyzed under the same conditions that induced mPTP opening. When human spermatozoa were exposed to 3 mmol/L of H_2_O_2_ for 60 min, an increase in PS externalization (77.50% ± 10.14) was observed in human spermatozoa compared to the untreated control (1.9% ± 1.8; *p* < 0.001; Figure 6A–D). Similarly, an increase in DNA fragmentation (2.31% ± 1.85) was observed after H_2_O_2_ exposure in human spermatozoa compared to the untreated control (0.6% ± 0.4; *p* < 0.05; Figure 6B–D). The increase in PS externalization and DNA fragmentation was accompanied by a decrease in sperm viability (50.4% ± 15.4) compared to the untreated control (94.8% ± 2.4; *p* < 0.05; Figure 6C), indicating that cell death processes were also activated in sperm cells under exogenous oxidative stress.

## 4. Discussion

Our study demonstrates for the first time that H_2_O_2_-induced exogenous oxidative stress causes opening of the mPTP, which is associated with mitochondrial dysfunction, alteration of sperm motility, and the expression of apoptotic cell death markers, including PS externalization and DNA fragmentation. The opening of the mPTP observed in the present work agrees with studies that have demonstrated the ability of H_2_O_2_ to induce this phenomenon in somatic cells [82,83,84,85,86,87,88]. The evidence indicates that activation of mPTP opening depends not only on ROS signaling, but also on other factors, such as disruption of Ca^2+^ homeostasis [44,89], being both second messengers and the main activators of mPTP opening in somatic cells [41,42]. Ca^2+^ signaling pathways in the cell are capable of interacting closely with ROS signaling, including H_2_O_2_, so that alterations in both Ca^2+^ homeostasis and ROS levels can affect each other, thus enhancing the harmful effects that can contribute to the pathogenesis of various disorders [44]. H_2_O_2_ causes the mobilization of Ca^2+^ from intracellular stores, increasing the concentration of Ca^2+^ in different cell types, including human spermatozoa [51,52,53,54,62,90], supporting the idea of the bidirectional interaction between ROS and Ca^2+^ signaling [91] as an important cellular signaling network [44], so alterations in one of the two systems could contribute to mitochondrial dysfunction [89]. A sustained increase in the concentration of Ca^2+^ within the mitochondrial matrix can induce the prolonged opening of the mPTP, which results in a disruption of mitochondrial function, leading to mitochondrial dysfunction and ultimately cell death [92,93]. However, the role of dysfunctional mitochondria in defective sperm has rarely been analyzed [35,94]. A sustained increase in the concentration of Ca^2+^ was observed under our experimental conditions, confirming the ability of H_2_O_2_ to mobilize Ca^2+^ from intracellular stores in human sperm. The Ca^2+^ mobilization can be explained by the ability of H_2_O_2_ to act as some hormones, neurotransmitters and growth factors, capable of activating phospholipase C of the plasma membrane, which, through the generation of inositol-1,4,5-triphosphate, facilitates the entry of Ca^2+^ through the plasma membrane [95] and stimulates the release of Ca^2+^ from intracellular stores [62]. The sustained increase in Ca^2+^ stimulates its accumulation in the mitochondrial matrix, leading to an overload of mitochondrial Ca^2+^, causing inflammation of the mitochondrial matrix, opening of mPTP [63] and release of pro-apoptotic molecules that lead to cell death [48]. This cell death caused by oxidative stress has been also associated with the mitochondrial membrane permeation [96], originating from mPTP opening [97], causing the dissipation of ΔΨm, reduced ATP levels, mitochondrial matrix swelling and ending with the rupture of the outer mitochondrial membrane [42] due to the opening of the mPTP [98,99]. Thus, both the dissipation of ΔΨm and the decrease in ATP levels observed in our study could be attributed to the opening of the mPTP. This causes a mitochondrial bioenergetics collapse, characterized by ΔΨm dissipation and a decrease in the metabolic rate by ATP production, explained because ATP synthase needs intact ΔΨm to generate ATP during OXPHOS [100,101]. On the other hand, the depletion of ATP induced by the opening of mPTP has been also associated with i) the permeabilization of the outer mitochondrial membrane that can cause the gradual release of components of the respiratory chain of the mitochondria, the dissipation of ΔΨm, which leads to a decrease in ATP generation and eventually to intrinsic apoptosis, or ii) the loss of ionic homeostasis and cellular integrity, ultimately resulting in necrosis [96,102,103]. The most frequently apoptotic markers in human spermatozoa are caspase activation and PS externalization [94,104]. PS externalization is an extremely important process in cells undergoing apoptotic cell death processes because it stimulates phagocytic activity through various bridging molecules and receptors [105]. Recently and under the same experimental conditions of concentration and time of exposure to H_2_O_2_ used in the present work, an increase in PS externalization was found [74], suggesting the first mechanism. Our results corroborate that exposure to H_2_O_2_ induces an increase in PS externalization and show for the first time that exogenous oxidative stress induced by H_2_O_2_ causes the activation of apoptotic-like phenomena through the mechanism that involves the opening of the mPTP in human spermatozoa.

Another feature of cell death previously observed in human spermatozoa after exposure to H_2_O_2_ is DNA fragmentation [61,106,107,108], coinciding with our study. It has been suggested that DNA fragmentation may be a consequence of the mitochondrial release of pro-apoptotic molecules, such as Endonuclease G (EndoG) and Apoptosis-inducing factor (AIF) [109]. Although it has been possible to detect the presence of EndoG in human spermatozoa, the physical architecture of the male gametes makes it difficult for these molecules to access the nuclear compartment [94,110]. The opening of the mPTP is one of the main pathways involved in the ability of sperm mitochondria to generate ROS and induce apoptotic cell death in sperm (reviewed in [64]). Therefore, the DNA fragmentation observed here, although low, may be directly associated with the increase in mROS, in agreement with the reports that most of the DNA damage observed in mature human sperm cells results from oxidative stress [65,111,112]. In spermatozoa, the mitochondrial functions, mainly the Ca^2+^ homeostasis and production of ROS and ATP, regulate processes such as motility, hyperactivation, capacitation, the acrosome reaction and fertilization [29,31,113,114,115]. The mitochondrial bioenergetics functions in the spermatozoa are responsible for the production of ATP through the OXPHOS pathway, fundamental in the viability and motility of the spermatozoa [116]. Therefore, sperm motility, a key function of the male gamete (reviewed by [117,118]), has been negatively related to impaired mitochondrial function and oxidative stress [119]. In this study, decreased motility was observed in sperm exposed to H_2_O_2_, in agreement with previous studies that showed altered sperm motility in sperm exposed to H_2_O_2_ [55,57,60,120,121]. Functional mitochondria are biomarkers of sperm quality, since the motility and fertilizing capacity of sperm are closely related to mitochondrial function in various species [122,123,124], and in human spermatozoa, the ΔΨm and respiratory efficiency have also been correlated positively with motility [125,126]. However, oxidative stress has been shown to cause alteration of mitochondrial function through dissipation of ΔΨm, uncoupling of OXPHOS, and subsequent decrease in ATP synthesis in sperm [127,128,129]. This allows us to explain our results and affirm that the decrease in motility in sperm exposed to H_2_O_2_ can be a consequence of the opening of the mPTP, the dissipation of ΔΨm and the reduction in ATP content, an essential factor in the maintenance of sperm motility [126].

## 5. Conclusions

H_2_O_2_-induced exogenous oxidative stress causes mitochondrial function damage associated with an increase in the intracellular Ca^2+^ concentration that induces the mPTP opening, ΔΨm dissipation, a decrease in ATP levels and mROS production. Furthermore, this study confirms the ability of human spermatozoa to undergo a cell death process with characteristics of apoptosis, since the alteration of mitochondrial function induced by H_2_O_2_ caused the expression of cell death markers such as PS externalization and DNA fragmentation, which may explain the loss of sperm function in patients with infertility associated with oxidative/nitrosative stress. The alteration of mitochondrial function finally leads to the alteration of sperm function, negatively affecting sperm motility, and therefore, the fertilizing capacity of the spermatozoon. The characterization of this mechanism of mitochondrial dysfunction and its relationship with the induction of cell death and the alteration of sperm function needs to be deepened in human spermatozoa. Further studies might be focused on developing strategies to protect the mitochondria and prevent this mechanism of damage induced by oxidative stress, which might benefit male patients facing decreased sperm quality associated with seminal oxidative stress.

## Data Availability

The data supporting the findings of this study are available from the corresponding author upon reasonable request.
